# Exploring Factors Influencing Cervical Cancer Screening Participation among Singaporean Women: A Social Ecological Approach

**DOI:** 10.3390/cancers16203475

**Published:** 2024-10-14

**Authors:** Qing Huang, Li-Ying Tan

**Affiliations:** Research & Data Analytics, Singapore Cancer Society, Singapore 168583, Singapore; liying_tan@singaporecancersociety.org.sg

**Keywords:** cervical cancer screening, social ecological model, Singapore, cancer information seeking, self-sampling, screening awareness, screening barriers, primary care providers’ role in screening

## Abstract

**Simple Summary:**

Cervical cancer poses a major public health concern in Singapore, but it can be effectively prevented through cervical cancer screening. Unfortunately, many Singaporean women have never been screened. Our research examined factors influencing screening participation in cervical cancer screening. We analyzed data from 665 Singaporean women, examining how knowledge, beliefs, health practices, and interpersonal and healthcare system influences affected their screening participation. Our findings showed that Malay women, those aged 25–29 years, and unmarried individuals were less likely to undergo screening. Conversely, women were more likely to undergo screening if they had better cervical cancer screening knowledge, understood that primary care doctors could perform the screening, had prior experience seeking cancer information, or were open to self-sampling options. These results shed light on potential multifaceted approaches to increase cervical cancer screening uptake, ultimately improving women’s health in Singapore.

**Abstract:**

**Background/Objectives**: Cervical cancer screening uptake in Singapore remains suboptimal. This study employed the Social Ecological Model (SEM) to investigate factors influencing cervical cancer screening participation among Singaporean women. **Methods**: The study included 665 women, aged 25–69 years, who reported awareness of cancer screening and no personal cancer history. Data were collected through a previously described online survey. Hierarchical logistic regression analysis was conducted to identify significant factors influencing screening participation. **Results**: Only 30% of participants reported cervical cancer screening participation. Women aged 25–29 years (OR = 0.33; 95% CI = 0.12–0.77), Malay women (OR = 0.42; 95% CI = 0.20–0.83), and unmarried women (OR = 0.30; 95% CI = 0.18–0.48) were less likely to be screened. Positive associations with screening participation were observed with good cervical cancer screening knowledge (OR = 2.90; 95% CI = 1.96–4.32), awareness of primary care providers’ role in delivering screening services (OR = 1.94; 95% CI = 1.24–3.10), cancer information seeking behavior (OR = 1.59; 95% CI = 1.07–2.39), and acceptance of self-sampling options (OR = 1.81; 95% CI = 1.22–2.70). **Conclusions**: Our study highlights the cumulative impact of factors at various SEM levels on screening participation and underscores the necessity for more targeted and multi-pronged strategies to improve cervical cancer screening uptake in Singapore.

## 1. Introduction

Cervical cancer is the fourth-most prevalent cancer affecting women globally, continuing to pose a substantial challenge to healthcare systems worldwide [1]. Persistent infection with high-risk types of human papillomavirus (HPV) is the primary risk factor for cervical cancer [2]. Other significant risk factors include having multiple sexual partners, smoking, early age at first pregnancy, and immunosuppression [3]. Early detection through cervical cancer screening is vital for effective prevention. The primary screening methods employed are the Papanicolaou (Pap) smear test, which detects abnormal cellular changes, and HPV testing, which identifies the presence of high-risk HPV types [2]. Many countries have implemented organized screening programs that typically integrate these two methods in age-specific strategies, thereby maximizing detection rates while minimizing unnecessary interventions [4].

In Singapore, despite a significant decline in incidence rates over the years (from 18.0 to 6.8 per 100,000 population between the periods 1968–1972 and 2017–2021), the reduction has slowed, and the proportion of cases diagnosed at advanced stages has risen in recent decades [5,6]. Additionally, cervical cancer imposes considerable economic costs in Singapore, with an estimated direct expense of SGD 57.6 million to the healthcare system over a 25-year period starting in 2008 [7]. These trends highlight the persistent burden of cervical cancer despite its high preventability through early detection and emphasize the need for enhanced prevention efforts. Singapore national guidelines advise women aged 25–69 years to undergo cervical cancer screening once every 3 or 5 years, depending on their age [8]. National screening programs and initiatives, such as “Screen for Life” and “Healthier SG” [9], were implemented to encourage more Singaporeans to go for subsidized screenings and follow-ups. Cervical cancer screening services are widely available at primary care providers (PCPs), including public polyclinics and private general practitioner (GP) clinics. However, despite longstanding efforts to provide subsidies and enhance accessibility to screening services, the cervical cancer screening uptake remains below target levels [10]. The National Population Health Survey 2022 reported suboptimal cervical cancer screening rates, with only 43.1% participation among women aged 25–74 years [11]. This rate lagged behind many countries with population-based cervical cancer screening programs [12,13,14], highlighting the need for improved strategies to enhance participation.

Cervical cancer screening uptake is influenced by a myriad of factors across different populations, such as socioeconomic status, knowledge, health behaviors, social supports, as well as healthcare system characteristics [15,16,17]. Many existing studies examining factors influencing cervical cancer screening behaviors predominantly utilize the Health Belief Model (HBM) [18,19,20]. The HBM is a psychological model that explains health-related behaviors by focusing on individuals’ beliefs and perceptions about health risks and the benefits of taking action [21]. While HBM provides valuable insights into individual-level determinants of screening behaviors, a more comprehensive framework is needed to include other important factors at interpersonal and broader influences, such as family and workplace influences, and healthcare services credibility and accessibility. Emerging in the late 1970s, the Social Ecological Model (SEM) offers a conceptual framework that incorporates these multiple levels of factors, typically categorized into individual, interpersonal, organizational, community, and policy levels [22]. Central to the SEM is the concept that health behaviors result from complex interplay among these various levels, emphasizing that individual health decisions are affected by broader environmental and social contexts. This approach has been employed in various research investigating the adoption of health behaviors [23,24], as well as studies on healthy lifestyle practices [25,26] and cancer screening uptake [27,28,29].

**Figure 1:**
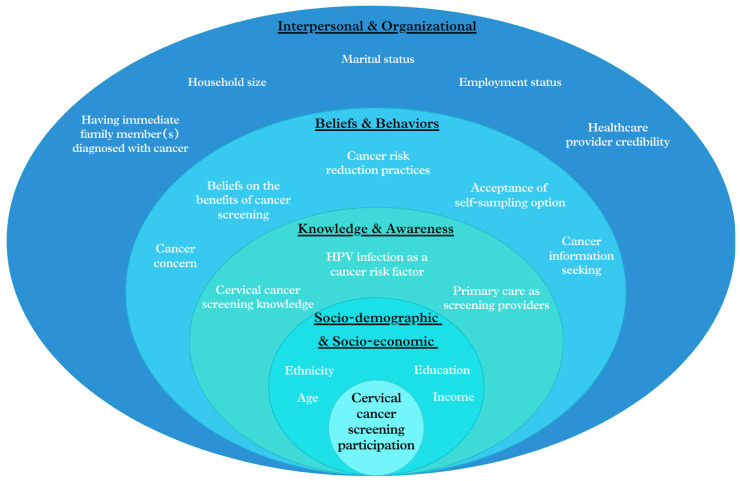
Conceptual framework of factors affecting participation in cervical cancer screening, based on the SEM approach.

Given the evolving healthcare landscape in Singapore, especially the growing involvement of PCPs in national screening initiatives, the SEM approach is particularly pertinent for exploring the multi-faceted factors affecting cervical cancer screening participation. By applying this multi-level approach, as illustrated in Figure 1, we aim to identify independent predictors of screening participation and examine the contributions of various factors ranging from individual knowledge, beliefs and behaviors to interpersonal and organizational influences. This study seeks to provide insights for developing more effective, multi-faceted strategies to enhance cervical cancer screening rates and improve relevant public health outcomes in Singapore.

## 2. Materials and Methods

### 2.1. Study Population

This study analysed a subset of data from a previously reported cross-sectional online survey [30]. In brief, the survey recruited 2000 Singapore citizens and permanent residents aged 21–69 years via the online panel of Toluna, a market research company (www.toluna.com). Participants were screened to ensure their eligibility, with implemented quotas for age, ethnicity and gender to maintain demographic representation. Upon survey completion, participants received incentives in the form of redeemable “Toluna points”.

This analysis was restricted to women aged 25–69 years, corresponding to the target population for cervical cancer screening in Singapore. Individuals who had personal cancer history, were unaware of cancer screening, or provided incomplete survey responses were excluded from the analysis. The final cohort comprised 665 eligible participants who met the above study criteria.

### 2.2. Variables

Table 1 provides a summary of all variables utilized in this study, detailing their measurements and categories.

The outcome variable, cervical cancer screening participation, was assessed using the question “Have you ever gone for cervical cancer screening?”. Participants were categorized as “ever-screeners” if they responded “Yes”, and “never-screeners” if they responded “No”.

Independent variables were grouped into four categories: (1) socio-demographic and socio-economic variables, (2) knowledge and awareness variables, (3) beliefs and behaviors variables, and (4) interpersonal and organizational variables.

### 2.3. Statistical Analysis

Descriptive statistics were employed to summarize participants’ socio-demographic and socio-economic characteristics, screening knowledge and awareness, health beliefs and behaviors, interpersonal and organizational factors, as well as the participation of cervical cancer screening.

Univariable analysis was conducted to assess the unadjusted relationships between potential influencing factors and cervical cancer screening participation. Variables with *p* < 0.2 in univariable analysis were incorporated into a hierarchical logistic regression. A series of nested models were constructed and compared to determine the added explained variance by each variable block:

(1) Model 1: Included socio-demographic and socio-economic variables

(2) Model 2: Added knowledge and awareness factors to Model 1

(3) Model 3: Added beliefs and behaviors variables to Model 2

(4) Model 4: Added interpersonal and organizational factors to Model 3

Odds ratios (OR) with corresponding 95% confidence intervals (CI) were reported for each variable in the models, and statistical significance was defined as *p* < 0.05. Model performance was evaluated using Akaike Information Criterion (AIC), −2 log likelihood, Nagelkerke’s R^2^, and chi-square. The change in explained variance (ΔR^2^) was calculated for each model to assess the incremental contribution of each variable block.

All statistical analyses were performed using R Statistical Software (version 4.3.3; R Foundation for statistical Computing, Vienna, Austria) [32].

## 3. Results

### 3.1. Participant Characteristics

Table 2 illustrates the characteristics of the study population, consisting of 665 women with an average age of 45.8 (12.4) years. The majority of participants were of Chinese ethnicity (75%), married or ever married (70%), employed (84%), and living in households with more than two family members (78%). Over half of the participants (57%) had attained tertiary education or higher. In terms of household income, 44% of participants were in the medium-high to high income categories. Notably, about one third of participants (31%) reported having immediate family members diagnosed with cancer.

### 3.2. Differences in Variables That Affect Participation of Cervical Cancer Screening

Table 2 shows that 30% of participants reported undergoing cervical cancer screening, representing less than one third of the study population.

Analysis of socio-demographic and socio-economic factors revealed significant differences between participants who had undergone screening (ever-screeners) and those who had not (never-screeners). Ever-screeners had notably lower percentages of individuals aged 25–29 years (3% vs. 14%), Malay women (6% vs. 14%), and low-income participants (12% vs. 19%) compared with never-screeners. However, educational attainment did not significantly differ between the two groups.

Regarding knowledge and awareness variables, while knowledge of HPV infection as a cancer risk was similar between ever-screeners and never-screeners, ever-screeners demonstrated higher levels across several key areas. As compared with never-screeners, ever-screeners exhibited better understanding of both Pap tests (95% vs. 60%) and HPV tests (46% vs. 28%) as cervical cancer screening modalities, and the correct age to begin screening (52% vs. 32%). This translated to a significantly higher percentage of ever-screeners with good overall knowledge of cervical cancer screening (70% vs. 41%). Furthermore, ever-screeners showed greater awareness of PCPs’ role in delivering cancer screening services, with 82% recognizing GPs and polyclinics as cancer screening providers, compared to 68% of never-screeners.

Among the variables related to beliefs and behaviors, a higher proportion of ever-screeners held favorable attitudes toward cancer screening, such as screening benefits of improving treatment outcomes (94% vs. 86%) and reducing mortality rates (84% vs. 77%). Acceptance of self-sampling was notably higher among ever-screeners compared to never-screeners (68% vs. 44%). Similarly, more ever-screeners reported engaging in healthy lifestyles to reduce cancer risks, including eating healthily (68% vs. 63%), exercising regularly (67% vs. 61%), and maintaining healthy weight (72% vs. 64%). Significantly more ever-screeners sought cancer information compared to never-screeners (70% vs. 54%). On the other hand, both groups exhibited similar levels of cancer-related concerns and shared comparable views on the screening benefit in terms of reducing treatment costs.

Regarding interpersonal and organizational variables, more ever-screeners reported being married or ever married (86% vs. 63%) and resided in households with more than two family members (85% vs. 75%) compared to never-screeners. However, employment status and perceived healthcare provider credibility showed no significant differences between ever-screeners and never-screeners.

### 3.3. Factors Predicting Cervical Cancer Screening Participation

The outcomes of hierarchical logistic regression analysis are presented in Table 3.

Model 1, which included only socio-demographic and socio-economic variables, explained 7% of the variance of cervical cancer screening participation. Compared to women aged 50–69 years, those aged 25–29 years showed notably lower odds of getting screened (OR = 0.22; 95% CI = 0.09–0.48). Malay women were half as likely to undergo screening compared to non-Malay women (OR = 0.44, 95% CI = 0.22–0.82). Conversely, women with higher incomes showed increased likelihood of undergoing screening compared to their low-income counterparts (OR = 1.87, 95% CI = 1.01–3.51 for the medium-high-income group; OR = 1.95, 95% CI = 1.09–3.56 for high-income group).

Model 2, which added knowledge and awareness variables to Model 1, increased the explained variance in cervical cancer screening participation from 7% to 18%. Good knowledge level (OR = 3.25; 95% CI = 2.26–4.71) and awareness of PCPs’ role in delivering cancer screening services (OR = 1.99; 95% CI = 1.30–3.09) were positively correlated with screening participation.

Model 3 incorporated belief and behavior variables alongside factors from Model 2, accounted for an additional 5% of the variance in cervical cancer screening participation. This model showed that individuals who reported ever seeking cancer information (OR = 1.69; 95% CI = 1.15–2.49) and those open to self-sampling options (OR = 1.83; 95% CI = 1.25–2.70) demonstrated higher likelihood of undergoing cervical cancer screening. Conversely, general beliefs towards cancer screening and self-reported cancer risk reduction practices did not demonstrate significant associations with screening participation.

**Figure 2:**
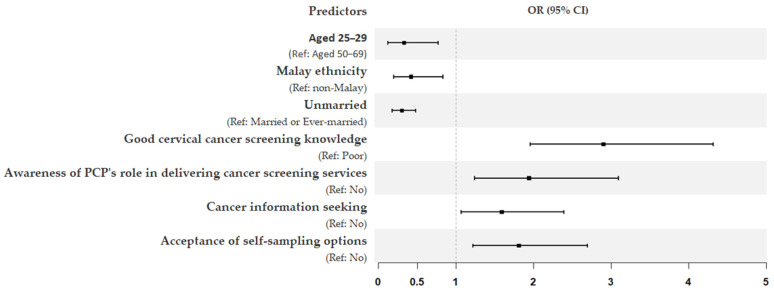
Forest plot summarizing final logistic regression model on the significant predictors influencing cervical cancer screening participation. The *x*-axis shows odds ratios (OR), and the error bars show 95% confidence intervals (CI).

Model 4, which further included interpersonal and organizational variables, increased the explained variance in screening participation to 28%. In this final model, unmarried women were three times less likely to undergo screening (OR = 0.30; 95% CI = 0.18–0.48). Several variables from previous models remained significant predictors of screening participation: age (25–29 years) (OR = 0.33; 95% CI = 0.12–0.77), Malay ethnicity (OR = 0.42; 95% CI = 0.20–0.83), good knowledge of cervical cancer screening (OR = 2.90; 95% CI = 1.96–4.32), awareness of PCPs’ role in delivering screening services (OR = 1.94; 95% CI = 1.24–3.10), cancer information seeking (OR = 1.59; 95% CI = 1.07–2.39), and acceptance of self-sampling options (OR = 1.81; 95% CI = 1.22–2.70). The forest plot in Figure 2 further provides a summary of all significant predictors influencing cervical cancer screening participation in this study.

## 4. Discussion

In 2020, the World Health Organization (WHO) initiated a global campaign aiming to eradicate cervical cancer as a public health concern by scaling up preventative, screening and treatment interventions [33]. In this context, our study observed a low screening participation rate of 30% among eligible participants in Singapore. This finding is particularly concerning given Singapore’s advanced healthcare system [34] and the well-established effectiveness of cervical cancer screening in reducing morbidity and mortality [35,36,37,38]. Comparatively, similar challenges in screening uptake have been observed across Asian countries, with participation rates mostly falling below 50% [39,40,41,42,43]. This similarity reflects shared regional barriers despite diverse cultural and economic contexts. In contrast, many other countries (such as the United States, Australia, and European countries) have achieved significantly higher screening rates of 70–80% [12,13,14,35]. These higher rates are largely attributed to integrated healthcare systems and well-established national screening programs supported with personal invitations, reminder systems, and extensive public health initiatives [4]. The disparity highlights the potential influence of cultural norms, healthcare system structures, and public health approaches on screening behaviors across different global regions. While the COVID-19 pandemic likely contributed partially to the low screening participation observed [44,45], this finding nonetheless highlights a critical gap in preventive healthcare utilization and underscores the pressing need for a more nuanced examination of factors affecting cervical cancer screening uptake within the Singaporean context, potentially drawing insights from successful global practices.

### 4.1. A SEM Perspective on Factors Influencing Screening Participation

Our study revealed a notable disconnect between participants’ general health practices and their engagement in cervical cancer screening. Despite widespread recognition on the advantages of cancer screening and adoption of healthy lifestyles to mitigate cancer risks, these attitudes and practices did not correlate with higher rates of cervical cancer screening participation. This finding highlights the complex nature of screening behavior and the need for targeted interventions. Employing the SEM framework, our hierarchical logistic regression analysis identified several significant predictors of screening participation across various levels. At the individual level, we found predictors such as participants’ knowledge of cervical cancer screening, awareness on PCPs’ role in delivering screening services, cancer information seeking behavior, and acceptance of self-sampling options. These are all highly modifiable factors, which could inform more targeted approaches in improving screening rates. Meanwhile, we also identified non-modifiable predictors at both individual and interpersonal levels, such as age, Malay ethnicity, and marital status. These non-modifiable factors offer crucial insights into tailoring strategies for specific demographic groups. While general health promotion efforts remain valuable, an SEM-informed strategy would be more effective in improving cervical cancer screening programs by simultaneously targeting modifiable and non-modifiable factors across multiple ecological levels, thereby creating more impactful interventions.

### 4.2. Key Factors Influencing Screening Participation

Numerous studies have identified individuals’ knowledge and awareness as crucial factors affecting screening uptake [16,42,46,47,48], and our study corroborate this. We found that, besides the knowledge gaps on cervical screening tests (especially HPV test) and appropriate age to start screening, there was insufficient awareness on the pivotal role of PCPs as screening providers, which was associated with screening participation. These findings align with and supports the current national initiative “Healthier SG”, which positions PCPs as central figures to holistically manage the health of residents, including regular health screening with fully subsidized services [9]. PCPs play crucial roles in the implementing primary prevention strategies and cancer screening protocols [49,50]. In light of these findings, it is paramount to enhance awareness of cervical cancer screening, especially among the eligible population. Educational campaigns and community outreach efforts should address these identified knowledge gaps, focusing on disseminating accurate information about cervical screening tests and the age to start screening, and highlighting the important role of PCPs in the screening process. Another key highlight could be the accessibility and affordability of screening through the “Healthier SG” initiative, emphasizing how the program makes preventative health checks convenient and cost-effective. This could help remove the traditional barriers of inconvenience and financial costs to cancer screening [51,52], making regular screening a practical option for all residents.

While knowledge and awareness are critical, they alone may not suffice to influence behavior [53]. Effectively translating knowledge and awareness into action often requires additional information or motivation. Our study consistently observed cancer information seeking as a crucial factor influencing cervical cancer screening participation, aligning with previous reports on the positive correlation between information seeking and screening uptake [54,55,56,57]. This behavior can influence screening participation through multiple pathways. For example, by actively seeking information, individuals may enhance their knowledge on screening tests and procedures, potentially mitigating anxiety on screening and overcoming perceived barriers such as embarrassment or time constraints. Access to accurate information can also reinforce positive outcome expectations and awareness of screening availability at PCPs. All these factors can be linked to higher screening participation directly or indirectly. Crucially, the quality and reliability of accessed information play a vital role in this process. Individuals who rely on more credible health information sources showed a higher likelihood of participating in effective cancer prevention and screening behaviors [58,59]. Our study showed that healthcare providers were cited as the most trusted source for cancer information. Given these results, while promoting health information seeking is generally beneficial, there is the need for targeted strategies to provide and guide individuals towards reliable and accurate sources of screening information. Enhancing healthcare providers’ role is crucial, given their high credibility level among patients [60] and the importance of patient-provider communication in promoting preventive health behaviors [61,62,63]. Healthcare providers should be equipped with professional tools and training, such as decision-aid-based counseling [64], to improve their ability to effectively communicate screening information and encourage screening participation beyond simply providing facts.

Emerging as a promising cervical cancer screening method, self-sampling addresses common screening barriers such as embarrassment, discomfort, and time constraints. By removing the need for gynecological visits and examinations, this method offers increased screening accessibility while maintaining similar sensitivity in screening outcomes [65,66]. Our findings demonstrated a positive association between acceptance of self-sampling options and cervical cancer screening participation. This correlation aligns with growing evidence, suggesting that self-sampling can enhance screening uptake, particularly among underserved populations [67,68]. The WHO has recommended self-sampling as an alternative method for cervical cancer screening [33]. Self-sampling has been integrated into national screening programs by many countries, such as the Netherlands, Denmark, and Australia [65]. Although not yet widely implemented in Singapore, a local study demonstrated high acceptability and ease of use of self-sampling, with 90% of participants expressing willingness to get screened if self-sampling were offered in future programs [69]. These findings are particularly relevant in light of our results showing lower screening rates among Malay women and unmarried women. Self-sampling could be a more acceptable and accessible screening option for these groups, given its potential to overcome cultural and personal barriers associated with traditional screening methods [70,71]. The implementation of self-sampling in Singapore may face challenges such as ensuring proper sample collection and addressing concern about test accuracy [68,72]. Nevertheless, introducing self-sampling could potentially enhance early detection of cervical cancer and precancerous lesions. Piloting self-sampling programs among cohorts with lower screening uptake could be a promising approach.

Our analysis revealed a marked underutilization of screening services among women aged 25–29 years, reaffirming findings from previous studies conducted in other countries [73,74,75]. This highlights a critical gap in screening coverage among younger women who have recently become eligible for cervical cancer screening. The lower screening rates in this age group may be attributed to several factors, including insufficient awareness on early screening, misconceptions about personal risk, and competing priorities such as career establishment or family commitments [76]. A recent Singapore study reported that women aged 25–29 years contributed 17% of all cervical intraepithelial neoplasia 2+ cases [77], which are precancerous changes that can develop into cervical cancer if not addressed. Importantly, early detection through screening and proper management of these precancerous changes greatly reduced the likelihood of progression to invasive cervical cancer [78,79]. Given the critical nature of early detection and specific challenges faced by this age group, targeted interventions should highlight the role of early screening in detecting precancerous lesions, as well as addressing relevant misconceptions [80], such as the notion that cervical cancer screening is not necessary following HPV vaccination. Given the digital savviness of this cohort, utilizing digital technologies for outreach, such as social media campaigns, or targeted online advertisements, could be particularly effective.

### 4.3. Hierarchical Analysis: Synthesizing Multi-Level Influences on Screening Participation

Employing the SEM framework, this study’s hierarchical logistic regression results demonstrate the cumulative impact of factors at different levels of influence on cervical cancer screening participation. The progressive increase in explained variance with each added factor set underscores the complex interplay of factors affecting screening behavior. Socio-demographic and socio-economic factors alone explained a relatively small proportion of the variance in cervical cancer screening participation, suggesting that interventions focusing solely on socio-demographic and socio-economic targeting may have limited effectiveness. The substantial increase when adding knowledge and awareness factors indicates that they play a key role in screening behavior, highlighting potential effectiveness of educational interventions in improving screening rates. The subsequent addition of beliefs and behaviors factors further improved the model, suggesting that comprehensive interventions addressing aspects of knowledge, beliefs and behaviors, which are all highly modifiable factors, could be more effective than knowledge-based approaches alone. The inclusion of interpersonal and organizational factors in the final model illustrates the importance of considering broader social and systemic influences on screening behavior, aligning with the SEM’s emphasis on multiple levels of influence. This also suggests that unaccounted factors at the community and policy levels may contribute to this complex health behavior, meriting further investigation in future studies.

### 4.4. Study Limitations and Future Directions

Several limitations of this study warrant consideration. First, the reliance on an online panel for recruitment might have introduced selection bias, potentially over-representing individuals with higher digital literacy and under-representing specific demographic groups. This could affect the generalizability of our findings to the broader Singapore population. Second, our evaluation on beliefs and attitudes towards cancer screening was generalized, and may vary across different types of cancer, potentially leading to unequal impacts on specific screening practices. Third, due to data availability constraints, we were unable to include societal and policy-level factors in our analysis. Future studies should address these limitations by adopting more diverse recruitment methods, addressing cervical cancer-specific beliefs and attitudes, and exploring broader societal and policy factors to gain a more comprehensive and deeper understanding of cervical cancer screening participation. While acknowledging these limitations, our study shed light on the diverse predictors of cervical cancer screening participation, from individual knowledge and behaviors to organizational influence. These findings highlight the necessity for more comprehensive and targeted strategies to enhance cervical cancer screening uptake in Singapore. Such initiatives could also potentially facilitate early detection, enhance treatment outcomes, and ultimately reduce the overall cervical cancer burden within the population.

## 5. Conclusions

In conclusion, our study revealed suboptimal cervical cancer screening participation in Singapore and identified factors influencing participation across various SEM levels. At the individual level, socio-demographic factors (such as age, Malay ethnicity), screening knowledge and awareness, health-related belief and behavior (such as acceptance of self-sampling options, cancer information seeking) significantly influenced screening participation. Marital status was a significant predictor at the interpersonal level. These findings demonstrate the utility of the SEM framework in understanding screening behaviors and underscore the multifaceted nature of factors affecting cervical cancer screening participation. Our results underscore the necessity for comprehensive, multi-level strategies to enhance cervical cancer screening uptake and improve public health outcomes in Singapore.

## Figures and Tables

**Table 1:** Summary of study variables and their measurements/categories.

Variable Category	Variables	Measurements/Categories
Outcome	Cervical cancer screening participation	No/Yes
Socio-demographic & socio-economic	Age	Years
	Age group	25–29/30–49/50–69
	Ethnicity	Chinese/Malay/Indian/Others
	Education	Secondary & below/Tertiary & above
	Household income ^1^	Low/Medium-low/Medium-high/High
Knowledge & awareness	HPV infection increases the chance of developing cancers	Unaware/Aware
	Knowledge level on cervical cancer screening ^2^	Poor/Good
	Awareness of PCPs’ role in delivering cancer screening services	No/Yes
Beliefs & behaviors	Cancer concern	Not concerned/Concerned
	Benefits of cancer screening ^3^	Disagree or Neutral/Agree
	Cancer information seeking	No/Yes
	Cancer risk reduction practices	No/Yes
	Acceptance of self-sampling options	No/Yes
Interpersonal & organizational	Marital status	Unmarried/Married or Ever married
	Household size ^4^	≤2/>2
	Employment status	Employed/Unemployed
	Having immediate family member(s) diagnosed with cancer	No/Yes
	Healthcare professionals as most reliable cancer information source ^5^	No/Yes

^1^ Household income categories were based on *Key Household Income Trends, 2019* [31]: low (below SGD 3000), medium-low (SGD 3000–SGD 8999), medium-high (SGD 9000–SGD 11,999), and high (SGD 12,000 and above). ^2^ Cervical cancer screening knowledge was assessed by three items based on national screening guidelines [8], covering the recommended initiation age and awareness of Pap and HPV tests as screening modalities. Participants received 1 point for each correct answer or reported awareness, with total scores (range: 0–3) categorized as “Poor” (<2) or “Good” (≥2). ^3^ Beliefs about the benefits of cancer screening were accessed through three questions as previously described [30], with responses dichotomized into “Disagree or Neutral” and “Agree”. ^4^ Household size was determined by the number of family members living in the same residence, which was classified into two groups: “≤2” and “>2”. ^5^ Healthcare provider credibility was assessed by participants’ selection of healthcare professionals as their most reliable cancer information source.

**Table 2:** Characteristics of participants by participation in cervical cancer screening.

	Overall (N = 665)	Cervical Cancer Screening Participation		*p*-Value ^2^
	n (%)	Never-Screener (N = 463) n (%)	Ever-Screener (N = 202) n (%)	
**Socio-demographic and socio-economic factors**				
Age ^1^	45.8 (12.4)	44.9 (12.7)	47.8 (11.2)	0.004
Age Group				<0.001
25–29	70 (11%)	63 (14%)	7 (3%)	
30–49	312 (47%)	213 (46%)	99 (49%)	
50–69	283 (43%)	187 (40%)	96 (48%)	
Ethnicity				<0.001
Chinese	502 (75%)	347 (75%)	155 (77%)	
Malay	79 (12%)	67 (14%)	12 (6%)	
Indian	56 (8%)	36 (8%)	20 (10%)	
Others	28 (4%)	13 (3%)	15 (7%)	
Education				0.50
Secondary and below	289 (43%)	205 (44%)	84 (42%)	
Tertiary and above	376 (57%)	258 (56%)	118 (58%)	
Household income				0.044
Low	113 (17%)	88 (19%)	25 (12%)	
Medium-low	265 (40%)	190 (41%)	75 (37%)	
Medium-high	124 (19%)	82 (18%)	42 (21%)	
High	163 (25%)	103 (22%)	60 (30%)	
**Knowledge and awareness factors**				
HPV infection increases the chance of developing cancers				0.79
Unaware	186 (28%)	131 (28%)	55 (27%)	
Aware	479 (72%)	332 (72%)	147 (73%)	
Pap test is the test used for cervical cancer screening				<0.001
Unaware	197 (30%)	186 (40%)	11 (5%)	
Aware	468 (70%)	277 (60%)	191 (95%)	
HPV test is the test used for cervical cancer screening				<0.001
Unaware	444 (67%)	335 (72%)	109 (54%)	
Aware	221 (33%)	128 (28%)	93 (46%)	
Cervical cancer screening should be started at the age of 25				<0.001
Unaware	411 (62%)	315 (68%)	96 (48%)	
Aware	254 (38%)	148 (32%)	106 (52%)	
Knowledge level on cervical cancer screening				<0.001
Poor	334 (50%)	273 (59%)	61 (30%)	
Good	331 (50%)	190 (41%)	141 (70%)	
Awareness of PCPs’ role in delivering cancer screening services				<0.001
No	186 (28%)	149 (32%)	37 (18%)	
Yes	479 (72%)	314 (68%)	165 (82%)	
**Beliefs and behaviors factors**				
Cancer concern				0.80
Not concerned	72 (11%)	51 (11%)	21 (10%)	
Concerned	593 (89%)	412 (89%)	181 (90%)	
Finding cancer early means less treatment costs				0.62
Disagree/Neutral	211 (32%)	150 (32%)	61 (30%)	
Agree	454 (68%)	313 (68%)	141 (70%)	
Finding cancer early means better treatment outcomes				0.008
Disagree/Neutral	76 (11%)	63 (14%)	13 (6%)	
Agree	589 (89%)	400 (86%)	189 (94%)	
Cancer screening is effective in reducing people’s risk of dying from cancer				0.064
Disagree/Neutral	138 (21%)	105 (23%)	33 (16%)	
Agree	527 (79%)	358 (77%)	169 (84%)	
Acceptance of self-sampling options				<0.001
No	324 (49%)	259 (56%)	65 (32%)	
Yes	341 (51%)	204 (44%)	137 (68%)	
Cancer information seeking				<0.001
No	275 (41%)	214 (46%)	61 (30%)	
Yes	390 (59%)	249 (54%)	141 (70%)	
Eating healthily to reduce cancer risk				0.18
No	236 (35%)	171 (37%)	65 (32%)	
Yes	429 (65%)	292 (63%)	137 (68%)	
Exercising regularly to reduce cancer risk				0.16
No	246 (37%)	179 (39%)	67 (33%)	
Yes	419 (63%)	284 (61%)	135 (67%)	
Maintaining healthy weight to reduce cancer risk				0.041
No	222 (33%)	166 (36%)	56 (28%)	
Yes	443 (67%)	297 (64%)	146 (72%)	
**Interpersonal and organizational factors**				
Marital status				<0.001
Unmarried	200 (30%)	171 (37%)	29 (14%)	
Married/Ever married	465 (70%)	292 (63%)	173 (86%)	
Household size				0.007
≤ 2	146 (22%)	115 (25%)	31 (15%)	
> 2	519 (78%)	348 (75%)	171 (85%)	
Employment status				0.90
Unemployed	107 (16%)	75 (16%)	32 (16%)	
Employed	558 (84%)	388 (84%)	170 (84%)	
Having immediate family member(s) diagnosed with cancer				0.044
No	461 (69%)	332 (72%)	129 (64%)	
Yes	204 (31%)	131 (28%)	73 (36%)	
Healthcare professionals as most reliable cancer information source				0.59
No	311 (47%)	213 (46%)	98 (49%)	
Yes	354 (53%)	250 (54%)	104 (51%)	
**Cervical cancer screening participation**	202 (30%)	**-**	**-**	

^1^ Mean (SD). ^2^ Wilcoxon rank sum test; Pearson’s Chi-squared test.

**Table 3:** Hierarchical logistic regression analysis of participation of cervical cancer screening.

Predictors	Model 1		Model 2		Model 3		Model 4	
	OR (95% CI) ^1^	*p*-Value	OR (95% CI) ^1^	*p*-Value	OR (95% CI) ^1^	*p*-Value	OR (95% CI) ^1^	*p*-Value
Age group (ref: 50–69)								
25–29	0.22 (0.09–0.48)	<0.001	0.19 (0.07–0.41)	<0.001	0.18 (0.07–0.42)	<0.001	0.33 (0.12–0.77)	0.016
30–49	0.85 (0.58–1.23)	0.39	0.80 (0.54–1.18)	0.26	0.80 (0.53–1.19)	0.27	0.94 (0.62–1.43)	0.77
Ethnicity (ref: non-Malay)								
Malay	0.44 (0.22–0.82)	0.014	0.54 (0.26–1.02)	0.07	0.58 (0.28–1.13)	0.13	0.42 (0.20–0.83)	0.017
Education (ref: Secondary and below)								
Tertiary and above	1.05 (0.71–1.53)	0.82	1.10 (0.73–1.64)	0.66	1.07 (0.71–1.62)	0.75	1.12 (0.73–1.71)	0.61
Household income (ref: Low)								
Medium-low	1.42 (0.84–2.45)	0.20	1.40 (0.81–2.48)	0.23	1.34 (0.77–2.40)	0.31	1.34 (0.75–2.43)	0.33
Medium-high	1.87 (1.01–3.51)	0.047	1.84 (0.97–3.55)	0.07	1.68 (0.87–3.30)	0.13	1.33 (0.67–2.67)	0.42
High	1.95 (1.09–3.56)	0.026	1.98 (1.08–3.72)	0.03	1.79 (0.96–3.41)	0.07	1.44 (0.75–2.80)	0.28
Knowledge level on cervical cancer screening (ref: Poor)								
Good			3.25 (2.26–4.71)	<0.001	2.74 (1.88–4.04)	<0.001	2.90 (1.96–4.32)	<0.001
Awareness of PCPs’ role in delivering cancer screening services (ref: No)								
Yes			1.99 (1.30–3.09)	0.002	1.89 (1.22–2.97)	0.005	1.94 (1.24–3.10)	0.004
Finding cancer early means better treatment outcomes (ref: Disagree/Neutral)								
Agree					1.53 (0.76–3.23)	0.25	1.55 (0.76–3.32)	0.24
Cancer screening is effective in reducing people’s risk of dying from cancer (ref: Disagree/Neutral)								
Agree					1.09 (0.66–1.84)	0.74	1.21 (0.72–2.07)	0.47
Cancer information seeking (ref: No)								
Yes					1.69 (1.15–2.49)	0.008	1.59 (1.07–2.39)	0.024
Acceptance of self-sampling options (ref: No)								
Yes					1.83 (1.25–2.70)	0.002	1.81 (1.22–2.70)	0.003
Eating healthily to reduce cancer risk (ref: No)								
Yes					0.89 (0.57–1.38)	0.59	0.88 (0.56–1.39)	0.60
Exercising regularly to reduce cancer risk (ref: No)								
Yes					1.03 (0.67–1.58)	0.90	0.98 (0.63–1.54)	0.93
Maintaining healthy weight to reduce cancer risk (ref: No)								
Yes					1.25 (0.79–1.98)	0.34	1.12 (0.70–1.81)	0.63
Marital status (ref: Married/Ever-married)								
Unmarried							0.30 (0.18–0.48)	<0.001
Household size (ref: ≤2)								
>2							1.55 (0.94–2.60)	0.089
Having immediate family member(s) diagnosed with cancer (ref: No)								
Yes							1.23 (0.82–1.85)	0.31
AIC ^2^	797.93		744.65		733.90		707.52	
−2 Log Likelihood	781.93		724.65		699.90		667.52	
Nagelkerke R^2^ (∆ R^2^)	0.07		0.18 (0.11)		0.23 (0.05)		0.28 (0.05)	
χ^2^	-		57.28		24.75		32.38	
*p*-value	-		<0.001		<0.001		<0.001	

^1^ OR = odds ratio, CI = confidence interval. ^2^ AIC = Akaike Information Criterion.

## Data Availability

The study data can be obtained from the corresponding author upon reasonable request.
